# The Joubert syndrome protein CEP41 is excluded from the distal segment of cilia in *C. elegans*

**DOI:** 10.17912/micropub.biology.000406

**Published:** 2021-06-07

**Authors:** Sebiha Cevik, Oktay I. Kaplan

**Affiliations:** 1 Rare Disease Laboratory, School of Life and Natural Sciences, Abdullah Gul University, Kayseri, Turkey

## Abstract

Rare diseases are a fundamental issue in today’s world, affecting more than 300 million individuals worldwide. According to data from Orphanet and OMIM, about 50-60 new conditions are added to the list of over 6,000 clinically distinct diseases each year, rendering disease diagnosis and treatment even more challenging. Ciliopathies comprise a heterogeneous category of rare diseases made up of over 35 distinct diseases, including Joubert syndrome (JBTS; OMIM 213300), that are caused by functional and structural defects in cilia. JBTS is an autosomal recessive condition characterized by a range of symptoms, including cerebellar vermis hypoplasia and poor muscle tone. There are now a total of 38 genes that cause JBTS, almost all of which encode protein products that are found in cilia and cilia-associated compartments, such as the basal body and transition zone. CEP41 is a JBTS-associated protein that is found in cilia and the basal body of mammals, but its localization in other ciliary organisms remains elusive. *C. elegans *is an excellent model organism for studying the molecular mechanisms of rare diseases like JBTS. We, therefore, decided to use *C. elegans* to identify the localization of CEP41. Our microscopy analysis revealed that CEPH-41(CEntrosomal Protein Homolog 41) not only localizes to cilia but is excluded from the distal segment of the amphid and phasmid cilia in *C. elegans*. Furthermore, we discovered a putative X-box motif located in the promoter of *ceph-41* and the expression of *ceph-41* is regulated by DAF-19, a sole Regulatory Factor X (RFX) transcription factor.

**Figure 1. CEPH-41 localizes to the amphid and phasmid cilia, but not in the distal segment of the amphid and phasmid cilia in C. elegans:**
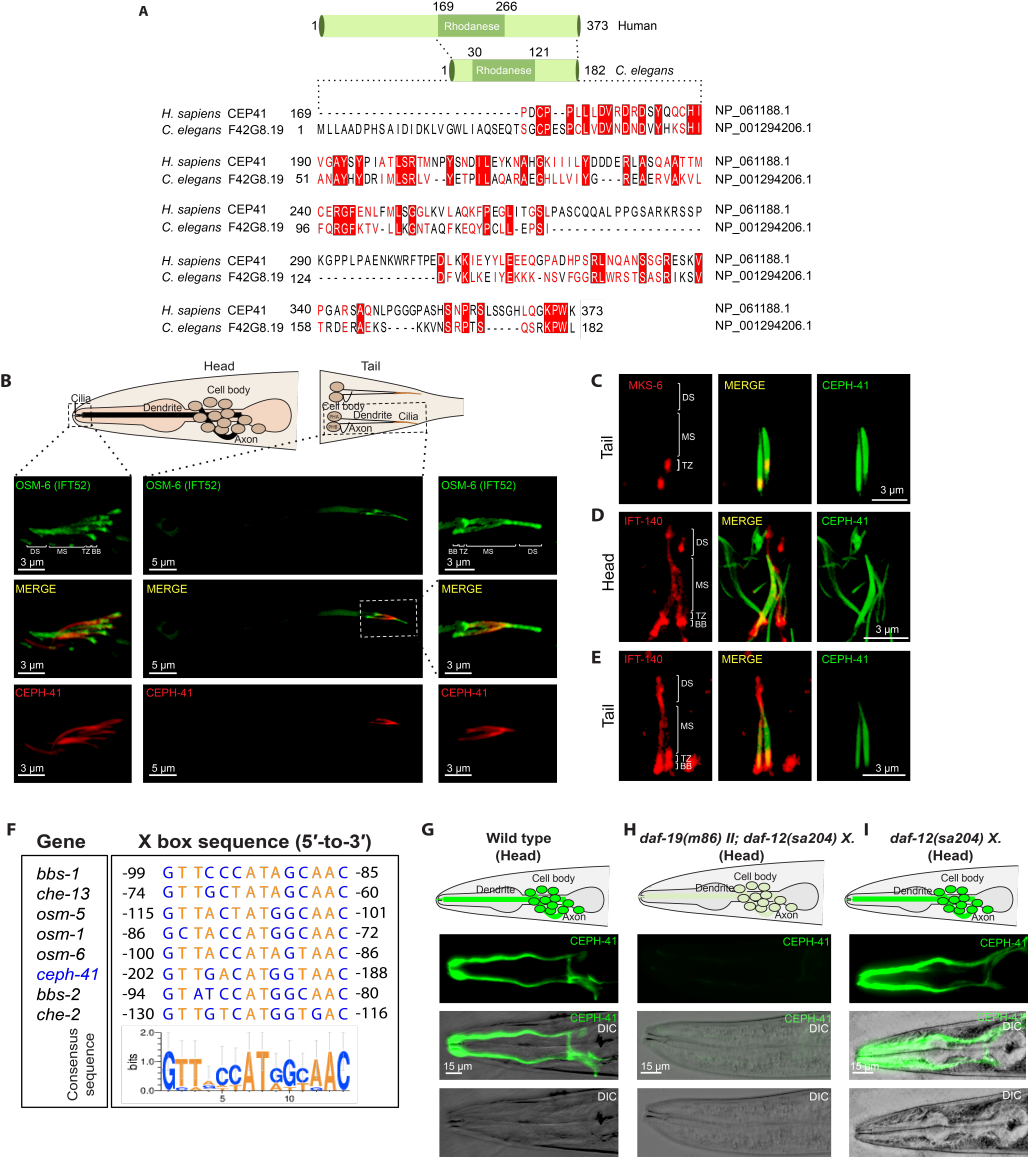
**A.** Schematic representation of human CEP41 and *C. elegans* F42G8.19 is displayed. The lengths of human CEP41 and *C. elegans* F42G8.19 are different, but they share the same protein domain (rhodanese). In both proteins, the positions of rhodanese domains are shown. The amino acid alignments of human CEP41 (169–373 aa) and *C. elegans* F42G8.19 (1–182 aa) are indicated. Identical and similar residues are displayed in white on red and red, respectively. **B.** Representative drawings of the ciliated sensory neurons found in the head (amphid) and tail (phasmid) of *C. elegans* are displayed. Dendrite, cell body (cell soma), axon, and cilia are shown. OSM-6::GFP (human IFT52) localizes to the whole ciliary axoneme in the head and tail while CEPH-41::wrmScarlet is concentrated in the proximal part of the cilium in the head and tail. DS, MS, TZ, and BB denote distal segment, middle segment, transition zone, and basal body, respectively. Scale bars are depicted at the left bottom of the images. **C. D. and E.** Co-localization of CEPH-41::GFP with IFT-140::mCherry (also known CHE-11) or MKS-6:: mCherry (human CC2D2A, a transition zone marker) was shown in the head and/or tails. IFT-140::mCherry (also known CHE-11) is an IFT protein that can be visible in the entire cilia. **F.** Shown is the comparative alignment of 14 bp X-box sequence motifs between ciliary genes and *ceph-41.* To display the sequence conservation of X-box nucleotide sequences, WebLogo was used to generate the graphical sequence logo. Each nucleic acid at different positions displays a particular frequency, which was reflected in the relative height of the corresponding nucleotide (Crooks, 2004). **G, H and I.** Representative single and merged images of the *C. elegans* head (amphid) were displayed for CEPH-41::GFP (green) and DIC in wild type, *daf-19(m86) II; daf-12(sa204) X.,* and *daf-12(sa204) X.* Scale bars, 15 µm.

## Description

We report the identification of *C. elegans* *F42G8.19* as the ortholog of human CEP41. This conclusion was based on database searches, reciprocal BLAST analysis, and cilia-specific localization. To look for the orthologous gene of human *CEP41* in *C. elegans,* we used threedatabases: the model organism Alliance of Genome Resources (https://www.alliancegenome.org/, Release 3.2.0), OrthoList 2 (http://ortholist.shaye-lab.org/, Release 2017), andConVarT (https://convart.org/, Release 2020) (Kim *et. al*., 2018; Pir *et. al*., 2021)*.* On the Alliance of Genome Resources and ConVarT, we discovered that *C. elegans* F42G8.19 is the ortholog of human CEP41. To validate this conclusion, we ran a manual reciprocal BLAST analysis (the Reciprocal Best Hits BLAST (RBHB))*.* The protein-protein BLAST (BLASTp) search of human CEP41 protein sequence (NP 061188.1) revealed F42G8.19 as the top *C. elegans* hit (Altschul *et. al*., 1990). In the following step, the protein sequence from the best hit *C. elegans* F42G8.19 (NP_001294206.1) was compared to human proteins, with human CEP41 emerging as the best match. *C. elegans* F42G8.19 encodes a 182-amino-acid protein that is shorter than the 373-amino-acid human CEP41 protein (**Figure 1A**). We subsequently performed the amino acid alignments of human CEP41 (169–373 amino acid) and *C. elegans* F42G8.19 (1–182 amino acid), which revealed over 24 % identity to each other (**Figure 1A**). We eventually determined whether *C. elegans* F42G8.19 has a rhodanese domain as human CEP41 (169-266 aa), and our query showed that *C. elegans* F42G8.19 has a rhodanese domain (30-121 aa) (**Figure 1A**) (Lee *et. al*., 2012). Taken together, our analysis revealed that *C. elegans* F42G8.19 is orthologous to human CEP41, and *C. elegans F42G8.19* was therefore assigned CEPH-41 (**CE**ntrosomal **P**rotein **H**omolog 41).

We generated a transgenic strain bearing the *ceph-41* promoter (1000 bp) together with a full-length of *ceph-41* (1173 bp) tagged withwrmScarlet, and determined the subcellular localization of *C. elegans* CEPH-41 (El Mouridi *et. al*., 2017). Our super-resolution confocal laser scanning microscope analysis revealed that CEPH-41::wrmScarlet is exclusively expressed in the ciliated sensory neurons and is localized to cilia in the head (amphid) and tail (phasmid), suggesting cilia localization of CEPH-41 is evolutionary conserved in both humans and *C. elegans* (**Figure 1B**) (Lee *et. al*., 2012). Additionally, our co-localization analysis showed that CEPH-41 is present in the transition zone (TZ) and the proximal cilia region known as the middle segment (the microtubule doublet containing segment) in *C. elegans*, but not in the distal cilia region (only A-tubule containing segment)(**Figure 1B and C**)*.* To independently validate the exclusion of CEPH-41 from the distal segment in the ciliated sensory neuron, we generated a new fluorescence marker for CEPH-41, tagging GFP to the C-terminus of *C. elegans* CEPH-41. A similar distal segment exclusion was detected when we examined the localization of CEPH-41::GFP (**Figure 1D and E**)*.* It would be interesting to check whether CEP41 would display a similar localization pattern in mammals and other organisms. CEPH-41 joins the club of middle segment localizing ciliary proteins, as another JBTS ARL-13/ARL13B protein is primarily enriched in the proximal cilia zone in some cells in both *C. elegans* (in amphid and phasmid cilia but not in AWB cilia) and mammals (MDCKII cilia, mouse oviduct, and tracheal tissue) (Cevik *et. al.,* 2010; Cevik *et. al.,* 2013; Li *et. al.,* 2010). Furthermore, CEP41 and ARL13B are both microtubule/tubulin-binding proteins that control ciliary tubulin glutamylation (Lee *et. al*., 2012; He *et. al*., 2018; Revenkova *et. al*., 2018; Gache *et. al*., 2010), but it is currently unknown how these two JBTS proteins are related. Finally, unlike ARL-13/ARL13B, which translocates in both directions along cilia, our time-lapse video analysis reveals that CEPH-41 does not appear to have IFT-like motility within cilia (Cevik *et. al.,* 2013).

Our current work reveals that CEPH-41 displays the mutually exclusive expression pattern in the ciliated sensory neurons, and the previous work already established that DAF-19, a sole Regulatory Factor X (RFX) transcription factor, is responsible for the mechanisms underlying ciliated-cell-specific expression of ciliary genes in *C. elegans.* The binding site for DAF-19 is the X-box sequence (13-15 base pair sequence) that is a regulatory motif in the promoter of cilia-specific genes in *C. elegans* (Blacque *et. al*., 2005; Chen *et. al*., 2006; Efimenko *et. al*., 2005; Phirke *et. al*., 2011; Swoboda *et. al.*, 2000). We next determined whether *ceph-41* has an X-box motif in its promoter. Scanning of the promoter of *ceph-41* (1000 bp upstream of the start codon) for the presence of an X-box motif revealed a putative X-box motif within the promoter of *ceph-41* (**Figure 1F**). We predict that ciliated-cell-specific expression of *ceph-41* is likely driven by DAF-19. We, therefore, crossed the transgenic strain expressing CEPH-41::GFP under its promoter into *daf-19* mutants and found that the ciliated-cell-specific expression pattern of CEPH-41 is dramatically reduced in *daf-19* mutants, suggesting the involvement of DAF-19 in the regulation of *ceph-41* expression (**Figure 1G, H and I**)

In conclusion, we present CEPH-41 as a ciliary protein that is absent from the distal segment of the amphid and phasmid cilia in *C. elegans*, and our future efforts are directed toward investigating the function of this middle segment protein in cilia in *C. elegans*.

## Methods

***C. elegans* strains, maintenance, and genetic cross**

The nematode growth medium (NGM) was used for culturing all *C. elegans*****strains at 20°C except for JT6924, *daf-19(m86) II; daf-12(sa204) X.* The JT6924 strain was cultured at 15°C in the NGM. The details of standard culturing protocols were described by Sydney Brenner in 1974 (Brenner, 1974). To generate *daf-19(m86)* expressing *N2;turEx16[CEPH-41p (F42G8.19)::CEPH-41::GFP::unc-54 3’UTR +rol-6],* we crossed *N2;turEx16[CEPH-41p (F42G8.19)::CEPH-41::GFP::unc-54 3’UTR +rol-6]* into *daf-19(m86) II; daf-12(sa204) X.* The *daf-12(sa204)* allele was used to prevent *daf-19(m86) II.* mutant worms entering into constitutive Daf phenotype (Daf-c) (Senti and Swoboda *et.al.,* 2008).

**Generation of transgenic worms by gonad microinjections**

1000 bp *F42G8.19* promoter together with entire *F42G8.19* coding sequence and introns were cloned and thus, two plasmids including *CEPH-41p (F42G8.19)::CEPH-41::GFP::unc-54 3’UTR* and *CEPH-41p (F42G8.19)::CEPH-41::wrmScarlet::unc-54 3’UTR* were generated. We generated transgenic worms expressing extrachromosomal arrays via microinjections. A mix of *CEPH-41p(F42G8.19)::CEPH-41::GFP::unc-54 3’UTR* (25 ng/μl) or *CEPH-41p (F42G8.19)::CEPH-41::wrmScarlet::unc-54 3’UTR* (1 ng/ μl) and the co-transformation marker *rol-6* plasmid (50 ng/ μl plasmid pRF4) were delivered to the gonads of 1-day adult worms with microinjections. In a brief, young adult worm (wild type) immobilized were placed onto a 2.5 % agarose pad with a small drop of Halocarbon oil (Sigma: 9002-83-9), where they were microinjected and recovered with a recovery buffer. For the microinjection, we used a Carl Zeiss Axio Vert.A1 Inverted microscope equipped with DIC optic and a Narishige Micromanipulator MMO-4. We transferred recovered P0 worms onto NGM plates containing OP50 bacteria and screened F1s with a behavioral roller phenotype under a stereotype microscope.

**Confocal Laser Scanning Microscopy for Analysis of Transgenic Strains**

The LSM900 confocal microscope with Airyscan 2 (ZEN 3 Blue edition software) was used to acquire high-resolution confocal images with a Plan ApoChromat 63x/1.40 NA objective. Microscope slides were prepared with a 2 % agarose pad for microscopy analysis and *C. elegans* were mounted on the agarose pad. 1-3 μL of 10 mM levamisole was applied to the middle of the agarose as an anesthetic agent. Confocal images with a Plan ApoChromat 63x/1.40 NA for one/two channels were obtained at intervals of 0.14 μm and these images were used to produce Z-stacks. A maximum intensity projection of the Z-stack images was processed with ZEN 3 Blue edition software, and the rest of the image analysis (rotation of images, arranging brightness, etc) was done with ImageJ (NIH) software (Schneider *et. al.,* 2012).

## Reagents

We would be happy to distribute strains, plasmids and annotated plasmid maps, and please email to oktay.kaplan@agu.edu.tr to request materials.

**Table:** 

**Strain**	**Genotype**	**Available from**
N2	*Caenorhabditis elegans*	CGC
JT6924	*daf-19(m86) II; daf-12(sa204) X.*	CGC
EJP81	*vuaSi24 [pBP43; Pche-11::che-11::mCherry; cb-unc-119(+)]II;* *unc-119(ed3) III; che-11(tm3433)V.* (CHE-11 is referred to as IFT-140 in the paper.)	Peterman Lab
	*vuaSi21[pBP39; Pmks-6::mks-6::mCherry; cb-unc-119(+)]II*	Peterman Lab
SP2101	*osm-6(p811); mnIs17[OSM-6::GFP; unc-36(+)*	CGC
OIK003	*N2;turEx16[CEPH-41p (F42G8.19)::CEPH-41::GFP::unc-54 3’UTR +rol-6]*	This study
OIK192	*N2;turEx20[CEPH-41p::CEPH-41 (F42G8.19)::wrmScarlet::unc-54 3’UTR +rol-6]*	This study
OIK156	*daf-19(m86) II; daf-12(sa204) X.;turEx16[CEPH-41p (F42G8.19)::CEPH-41::GFP::unc-54 3’UTR +rol-6]*	This study
OIK830	*osm-6(p811); mnIs17[OSM-6::GFP; unc-36(+)];turEx20[CEPH-41p::CEPH-41(F42G8.19)::wrmScarlet::unc-54 3’UTR +rol-6]*	This study
OIK1014	*N2;turEx16[CEPH-41p (F42G8.19)::CEPH-41::GFP::unc-54 3’UTR +rol-6]* *;vuaSi24 [pBP43; Pche-11::che-11::mCherry; cb-unc-119(+)]II; unc-119(ed3) III; che-11(tm3433)V.*	This study
OIK1084	*daf-12(sa204) X.;turEx16[CEPH-41p (F42G8.19)::CEPH-41::GFP::unc-54 3’UTR +rol-6]*	This study
OIK1091	*turEx16[CEPH-41p (F42G8.19)::CEPH-41::GFP::unc-54 3’UTR +rol-6]; Pmks-6::mks-6::mCherry; cb-unc-119(+)]II*	This study
**Plasmids**	**Genotype**	**Description**
OK41	*CEPH-41p::CEPH-41(F42G8.19)::wrmScarlet::unc-54 3’UTR*	This study
OK29	*CEPH-41p::CEPH-41(F42G8.19)::GFP::unc-54 3’UTR*	This study
pRF4	*rol-6(su1006)*	(Mello *et. al.,* 1991)
